# Screening for Copy Number Variations of the 15q13.3 Hotspot in *CHRNA7* Gene and Expression in Patients with Migraines

**DOI:** 10.3390/cimb43020078

**Published:** 2021-09-07

**Authors:** Mehmet Fatih Özaltun, Sırma Geyik, Şenay Görücü Yılmaz

**Affiliations:** 1 Department of Neurology, Gaziantep University, Gaziantep 27310, Turkey; dr.fozaltun@gmail.com (M.F.Ö.); drsirmageyik@hotmail.com (S.G.); 2 Department of Nutrition and Dietetics, Gaziantep University, Gaziantep 27310, Turkey

**Keywords:** DNA copy number variation, chromosome 15q13.3 microdeletion syndrome, migraine, chromosome deletion, cholinergic agents, biomarkers, gene dosage

## Abstract

Background: a migraine is a neurological disease. Copy number variation (CNV) is a phenomenon in which parts of the genome are repeated. We investigated the effects of the CNV and gene expression at the location 15q13.3 in the Cholinergic Receptor Nicotinic Alpha 7 Subunit (*CHRNA7*) gene, which we believe to be effective in the migraine clinic. Methods: we evaluated changes in *CHRNA7* gene expression levels and CNV of 15q13.3 in patients with migraine (*n* = 102, with aura, *n* = 43; without aura, *n* = 59) according to healthy controls (*n* = 120) by q-PCR. The data obtained were analyzed against the reference telomerase reverse transcriptase (*TERT*) gene with the double copy number by standard curve analysis. Copy numbers were graded as a normal copy (2), gain (2>), and loss (<2). Results: we analyzed using the 2^−^^ΔΔCT^ calculation method. The *CHRNA7* gene was significantly downregulated in patients (*p* < 0.05). The analysis of CNV in the *CHRNA7* gene was statistically significant in the patient group, according to healthy controls (*p* < 0.05). A decreased copy number indicates a dosage loss. However, no significant difference was observed among gain, normal, and loss copy numbers and expression values in patients (*p* > 0.05). The change in CNV was not associated with the downregulation of the *CHRNA7* gene. Conclusion: Downregulation of the *CHRNA7* gene may contribute to the formation of migraine by inactivation of the alpha-7 nicotinic receptor (α7nAChR). The association of CNV gains and losses with migraines will lead to better understanding of the molecular mechanisms and pathogenesis, to better define the disease, to be used as a treatment target.

## 1. Introduction

A migraine is a severe neurological disease; symptoms include recurring headaches, nausea, and vomiting [1]. The most common triggers of a migraine include stress, weather changes, menstruation, excessive sleepiness, or insufficient sleep [2].

Migraine symptoms can differ between patients. Various drugs are used to reduce these symptoms. These drugs do not cure migraines, but they can reduce the frequency of attacks or make them easier to overcome. In about half of the cases, migraines are hereditary [3]. This complex neurological disease is strongly influenced by both genetic and environmental factors, including hormones and stress. It has been reported that nitrosative and oxidative stress in platelets are particularly associated with migraine attacks [4]. Over the past decade, numerous studies have demonstrated the multifaceted polygenic character of this disease, with the identification of many candidate genes involved in migraine sensitivity, including vascular, neurotransmitter, and hormone-related genes [5].

CNVs are changes in the copy number (both an increase and a decrease) of any DNA segment present in an individual’s genome and may or may not contain a gene. Genetic variants, including splicing, deletion, and duplication of DNA segments, are collectively referred to as CNV. CNVs constitute an important part of the genetic variation between individuals. After the Human Genome Project was completed, the researchers found that the genome experienced gains and losses of genetic material. Although there are studies on the contribution of copy numbers to diseases [6], their relationship is not clear. However, it has been known for a long time that some cancers are associated with high copy numbers of certain genes and are called the “copy number variations”. CNVs differ between individuals. Copy number variation is a type of structural variation in which we have a three- or even four-fold DNA sequence in some individuals. When we look at the chromosomal region where they are located, we observe a change in the number of copies in normal people. Sometimes these copy number variants may contain one or more genes; These CNVs may contain genes or several genes. This means that an individual has four copies of that gene instead of two, another individual has three, or another individual has a different copy. In some cases, if these genes are involved in dose-sensitive functions [6] and they are tissue-specific [7], they may be directly related to disease risk or disease. Copy number variations are effective in multiple neuropsychiatric conditions, including Autism, schizophrenia, and intellectual disability (ID) [8]. Chromosome 15q13 is a hot spot for such CNVs [9]. Many varieties of these CNVs are over-represented in individuals with neuropsychiatric disorders). Dosing sensitivity of the *CHRNA7* gene, which is encoded for the α7 nicotinic acetylcholine receptor in the human brain, is the major driver of cognitive and behavioral phenotypes [10].

Nicotinic acetylcholine receptors represent the ligand-gated ion channel family and are members of the cys-loop receptor superfamily [11]. Although these ion channels have a high expression in the brain, *CHRNA7* expression is highest in the hippocampus [12]. Alpha 7 nAChRs, which take place both presynaptically and postsynaptically, are important in regulating both inhibitory and stimulating neurotransmitter release, as well as mediating signal transmission at synapses [13]. The chromosomal location of the *CHRNA7* gene is 15q13.3 (https://www.omim.org/entry/118511, accessed on 1 April 2021). 15q13.3 microduplication is a very rare genetic condition in which a small extra piece of one of the 46 chromosomes (chromosome 15) is found. This piece comes from the region on chromosome 15 known as q13.3. The extra piece of a chromosome is very small and called microduplication. Most of our knowledge about 15q13.3 microduplication was obtained from the examination of individuals who were referred for genetic testing. While a developmental delay, unusual behavior, or a health problem may be the cause of its detection, 15q13.3 microduplication has sometimes been detected in another member of the family [14]. In individuals with 15q13.3 replication, a small amount is repeated within the long arm (q) of chromosome 15. Humans with one copy of the 15q13.3 cytogenetic band typically have duplication about 1.5 million base pairs (Mb) [15]. When we look at the gene-related medical results, we first encounter microduplication syndrome as a rare chromosomal disorder. While some individuals with this duplication do not have any medical and behavioral problems, some individuals have developmental delay, intellectual disability, communication difficulties, behavioral and psychiatric problems (such as autistic features, emotional inability, attention deficit hyperactivity, schizophrenia), nutritional problems, insomnia, hypotonia, and seizures can be monitored [16]. The relationship between the number of copies in the *CHRNA7* gene and clinical severity is expressed according to the rating between 0 and 4 (0; homozygous deletion, 1; heterozygous deletion, 2; normal, 3; heterozygous duplication, 4; triplication or homozygous duplication). CNVs including *CHRNA7* are effective in neuropsychiatric diseases. Dosage sensitivity of *CHRNA7* results in more serious clinical and penetrant consequences due to the loss of the copy number [14].

Two general methods are used to reveal the diversity of CNVs in disease and health. The first technique is array-based competitive genomic hybridization (array-CGH). It uses the feature that any region that is duplicated or deleted in the sample of an individual has a similar DNA duplication or deletion to that region concerning the reference genome. This technique is called sequence-based competitive genomic hybridization (sequence-CGH) and is based on the property that any region amplified or deleted in an individual’s sample has a DNA duplication or deletion similar to that region in relation to the reference genome. Consequently, this method aims to detect these differences in relative DNA content. The second technique is binary end pairing. It does not detect direct copies and deletions, but instead detects length differences in the size of the gene from a sample relative to the reference genome. Parts that appear too large should contain duplication or insertion, while fragments that appear too large should contain deletions. Very large visible parts must include the replication or insertion; small parts must include the deletion. Methods such as quantitative PCR and fluorescent in situ hybridization can be used to verify CNVs [17]. The method that focuses on the relative comparison of DNA content (hybridization-based mapping) is based on microarray and detects copy number differences by allowing the fluorescently labeled DNA of a sample individual to hybridize to a designed sequence from different regions of the genome.

Changes in gene expression serve as a support for the complexity and diversity of human phenotypic variation, and therefore, a comprehensive study of the effect of CNVs on variation in gene regulation can have profound implications for our understanding of the genetic basis of complex traits [18]. Understanding the genetic basis of phenotypic variation in human populations is one of the main goals of human genetics today. Evidence shows that an increased copy number will help increase gene expression with positive expression [19,20] or negative gene expression levels (deletion of a transcriptional repressor), but the relative contribution of such large genetic variants (CNVs) and smaller variants, according to the phenotypic variation were not evaluated (SNPs) [21,22].

In this study, the expression of the CNVs in the *CHRNA7* gene at the 15q13.3 location in migraine patients and revealing the gene expression and clinical linkage, and the potential of CNVs and/or gene expression as a biomarker and a new target in the treatment of the disease was investigated.

## 2. Materials and Methods

### 2.1. Study Population

The patient group was composed of individuals who applied to Gaziantep University Faculty of Medicine, Department of Neurology, and were followed-up and diagnosed with migraines, according to The International Classification of Headache Disorders 3rd Edition (ICHD-3) diagnostic criteria. The control group consisted of healthy individuals who applied to the same clinic and did not have a family history of migraines or any neuropsychiatric disease. In the study, age, medical history (hypertension, diabetes mellitus, chronic lung disease, kidney disease, infections, stroke, endocrine disease, asthma), family history, drug use characteristics (naproxen, amitriptyline, moclobemide, citalopram, beta-blocker, valproic acid & topiramate, calcium-channel blockers, profen, cyproheptadine, triptan, paracetamol, ASA, selective serotonin reuptake inhibitors, diclofenac) were recorded. Total samples (*n* = 222) were comprised of migraine patients (*n* = 102) (female, *n* = 90; male, *n* = 12) (with aura, *n* = 43; without aura, *n* = 59), and healthy controls (*n* = 120) (female, *n* = 73; male, *n* = 47). The age range of the participants was between 19 and 60. Whole peripheral blood samples were taken from each individual into the routine blood tubes containing K3 EDTA. DNA and RNA samples were obtained from whole blood for genetic analysis. The consent was obtained from all individuals.

### 2.2. Selection of CHRNA7 Gene and CNV

Both presynaptic and postsynaptic α7nAChRs are important in regulating both inhibitory and stimulating neurotransmitter release, as well as mediating signal transmission at synapses. Presynaptic and postsynaptic α7nAChRs are important in regulating both inhibitory and excitatory neurotransmitter release, as well as mediating signal transduction at synapses; 15q13.3 microduplication in the gene is a very rare genetic condition. According to the literature research on the gene, some individuals with this duplication do not have any medical and behavioral problems, while some individuals develop retardation, intellectual disability, communication difficulties, behavioral, and psychiatric problems (autistic features, emotional inadequacy, attention deficit hyperactivity, schizophrenia), feeding problems, insomnia, hypotonia, and seizures were found [16]. With the loss or increase of the copy number that causes the dosage sensitivity of *CHRNA7*, more serious consequences may occur penetrant and clinically. α7nAChR activation can greatly reduce the neuroinflammation response. Neuroinflammation has an important role in the pathogenesis of chronic migraine (CM). In addition, clinical results show that nicotine gum causes analgesic effects in migraine patients [23]. However, the role of α7nAChR in migraine is unclear. The gene of interest was screened in OMIM (https://www.omim.org/entry/157300, accessed on 1 April 2021) and it was determined that there was a link between the 15q11-q13 (*MGR7*; 609179) region for *MGR7* (migraine with aura, susceptibility to, 7). Subsequently, CNVs on the gene were investigated in the SFARI database (https://gene.sfari.org/database/cnv/, accessed on 1 April 2021, latest release: 2020) [24]. Due to the proximity of these two locations, the 15q13.3 region was chosen because the CNVs in the region may be effective at the molecular basis of the migraine and there is no information on this subject in the literature (according to the authors’ information).

### 2.3. DNA and RNA Extraction

A total of 3 mL of whole blood samples were drawn into the tubes with EDTA, from each subject, for the *CHRNA7* gene CNV and expression analysis. Blood samples belonging to control and patient groups were stored at −20 °C until RNA and DNA were obtained. After the samples were brought to room temperature, a total of 1 mL of blood samples were used to obtain DNA (40 µg) and RNA (10 μg). DNA was extracted from whole blood with a DNA extraction kit (PureLink^TM^ Genomic DNA Mini Kit, K182002) (Carlsbad, San Diego, CA, USA). Total RNA was extracted from whole blood by modifying the acid guanidinium thiocyanate-phenol-chloroform (AGPC) RNA extraction method [25]. DNA and RNA quality was determined by spectrophotometer (Thermo Scientific, Multiskan GO) (Carlsbad, San Diego, CA, USA).

### 2.4. Complementary DNA (cDNA) Synthesis with Reverse Transcriptase PCR (RT-PCR)

RNA samples obtained were performed using an RT kit (QuantiTect^®^, Reverse Transcription Kit, Qiagen, 205313) (Germantown, MD, USA) according to the manufacturer’s protocol. A total of 20 µL of PCR reaction was prepared with a total of 10 ng of RNA, 5 × HiSpec Buffer, 10 × Nucleic Mix, Reverse Transcriptase Mix, and RNase-free water. The reactions were incubated at 16 °C for 30 min, 42 °C for 30 min, and 85 °C for 5 min using an automatic Thermal Cycler (Veriti, Applied Biosystems) (Foster, CA, USA) was then immediately placed on ice and stored at −20 °C until preparation of the gene expression.

### 2.5. Quantitative-Comparative CT (ΔΔCT) Real-Time PCR (qPCR) for Gene Expression

qPCR reactions were performed on a Rotor-Gene Q (Germantown, MD, USA). For *CHRNA7* gene expression standardization, β-Actin (ACTB) was used as endogenous control, and a reference human RNA was used as an RNA control (Life Technologies; AM7155) (Carlsbad, San Diego, CA, USA). A total of 25 µL PCR reaction, 5 µL RT-PCR products, 2× SYBR Green Universal PCR Master Mix (Qiagen) (Germantown, MD, USA), and 900 nM of each primer (*CHRNA7* and *ACTB*) (Qiagen) (Germantown, MD, USA) were prepared. Reactions were incubated at 96 °C pre-incubation, 95 °C for 10 min for 1 cycle. It was analyzed as 40 cycles of 15 s at 95 °C, 30 s at 55 °C, and 40 s at 70 °C. All reactions were carried out in triplicate, and 2^−^^ΔΔCT^ values were calculated [26].

### 2.6. Copy Number Variation (CNV) Analyses

Copy number variation was analyzed with Rotor-Gene Q using DNA samples prepared according to the kit protocol for *CHRNA7* (Thermo Fisher Scientific, 4400291) (Waltham, MA USA) and the reference *TERT* gene (Thermo Fisher Scientific, 4403316) (Waltham, MA USA). Real-time PCR analysis was prepared with 5.5 μL of 2 × master mix, 0.55 μL of 20 × TaqMan copy number Assay, 0.55 μL of 20 × TaqMan Copy Number Reference Assay, and 2.2 μL of RNAse-free water in a total reaction mixture of 8.8 μL. The prepared mixture was distributed to sample tubes and 8 µL of diluted DNA samples for each sample was added to 10 µL. The reaction was analyzed in real-time PCR (Rotor Gene-Q, Qiagen) for 10 min 1 cycle at 95 °C, 15 s at 95 °C, and 40 cycles for 60 s at 60 °C. The data were analyzed according to the reference *TERT* gene with two copy numbers by relative quantitation analysis and 2^−^^ΔΔCT^ values were calculated [26].

To determine the number of copies, a relative quantitation analysis was performed between the unknown sample (*CHNRA7*-FAM probe) and a calibrator sample (*TERT*-VIC probe). The sample was combined with both tests, master mix, and then repeated four times by real-time PCR. The *CHRNA7* gene was analyzed according to *TERT* (a reference sample known to have two copies of the test sequence) and the ΔCt value was calculated for both test samples and calibrator [26,27]. In the process, the finding of a copy of the *TERT* gene on the X chromosome in healthy male individuals confirms the analysis. The obtained data were compared with the RNA fold values compared to the reference gene, and the copy numbers were evaluated as the normal copy number: 2, increased 2> and decreasing copy number <2.

### 2.7. Statistical Analyses

The compliance of the data to normal distribution was tested with the Shapiro–Wilk test. The Mann–Whitney U test was used for the comparison of variables that did not have a normal distribution in two independent groups, and the Kruskal–Wallis and Dunn multiple comparison tests were used to compare more than two independent groups. The relationships between categorical variables were tested with the Chi-square test, and the odd’s ratio and 95% confidence intervals were estimated. As descriptive statistics, mean ± standard deviation for numerical variables and number and percentage (%) values for categorical variables are given. Using the SPSS package program (Release 22.0, SPSS Inc., Chicago, IL, USA) for statistical analysis, *p* < 0.05 was considered statistically significant. ROC curve analysis was used to evaluate the potential of *CHRNA7* copy number and the protein expression level of the gene as biological markers in migraine patients. SPSS package program (Release 22.0, SPSS Inc., Chicago, IL, USA) was used for analysis. Breakpoints are made according to Youden’s maximum index.

## 3. Results

### 3.1. Population Data

When the patients and controls were compared in terms of gender, alcohol and smoking, diabetes, and hypertension, the number of women and smokers in the migraine group was found to be statistically significant (respectively *p* = 0.001, *p* = 0.049). Clinical data were not significant, except for gender and smoking (Table 1).

When age, body mass index (BMI), white blood cell (WBC), urea, glomerular filtration rate (GFR), erythrocyte sedimentation rate (ESR), C-reactive protein (CRP), serial hemoglobin (HBG) values were analyzed together, WBC and HBG values were found to be lower (respectively *p* = 0.025, *p* = 0.027) and estrogen (ESR) values were found to be higher (*p* = 0.007) and statistically significant in the migraine group compared to the controls (Table 2).

### 3.2. CHRNA7 Gene Expression

The distribution of expression levels of the *CHRNA7* gene, which is in the genetic etiology of migraine disease, in the control and patient groups was examined and their *p*-values and expression levels were calculated (Table 3).

**Figure 1:**
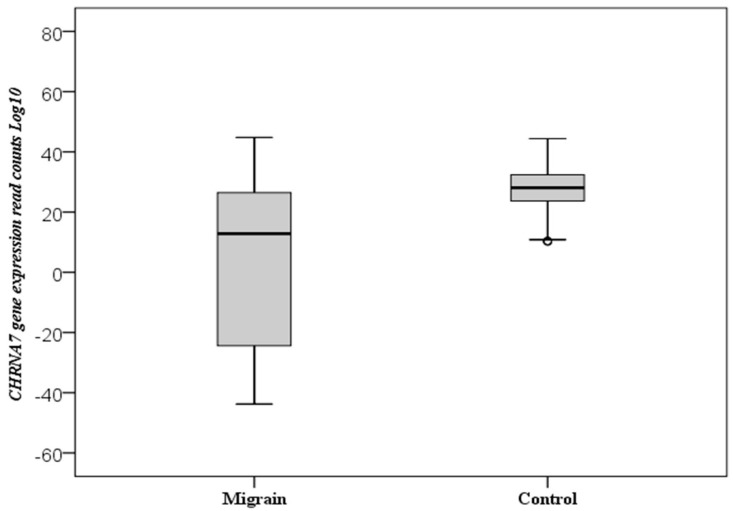
Boxplot of the *CHRNA7* gene expression. The total numbers of miRNA reads are log10 transformed. The circles represent outliers.

A statistically significant difference was observed between the control and patient groups in terms of expression levels of the *CHRNA7* gene (relative to the β-actin (*ACTB*) endogenous control gene), and *CHRNA7* gene expression values were found to be significantly lower in the migraine group (*p* = 0.001) (Figure 1).

### 3.3. CNV Measurements of CHRNA7 Gene

The distribution of copy number variation of the *CHRNA7* gene in the control and patient groups was examined, and their *p*-values and descriptive statistics were calculated. The copy number in the *CHRNA7* gene was observed as both a decreased and increased copy number in the patient group and was statistically significant compared to the controls (*p* = 0.001). The number of reduced CNVs (<2) in the *CHRNA7* gene was lower in patients with migraines than in the controls (Table 4). A reduced copy number in the gene indicates a deletion compared to the normal copy number.

### 3.4. Evaluation of CHRNA7 Gene Expression and Measurements of CNV

In the migraine patients and controls, the expression data of the *CHRNA7* gene compared to the *ACTB* control gene, and the relationship between copy number changes in the gene were examined; moreover, median and *p* values were calculated. Gene expression levels in both migraine and control groups were not statistically significant compared to the copy numbers (*p* > 0.050) (Table 5).

Although it was not statistically significant, considering the averages, differences were observed between CNVs and *CHRNA7* gene expression data. There was a significant decrease (4.27-fold) between the expression value (6.49 ± 27.94), and the expression value (27.73 ± 9.96) compared to controls (<2) when patients had reduced copy number (<2). However, no difference was observed between individuals with migraines in terms of the relationship among the decreased, normal, and increased copy numbers and expression values. It was determined that the change in CNV does not affect the expression of the gene.

### 3.5. The Clinical Variables and Measurements of CHRNA7 Gene Expression Levels

By examining the relationship between the clinical variables of migraine patients and the measurement of *CHRNA7* gene expression levels, mean, median, and *p*-values were calculated. The relationship between clinical variables and *CHRNA7* gene expression was evaluated (Table 6).

**Figure 2:**
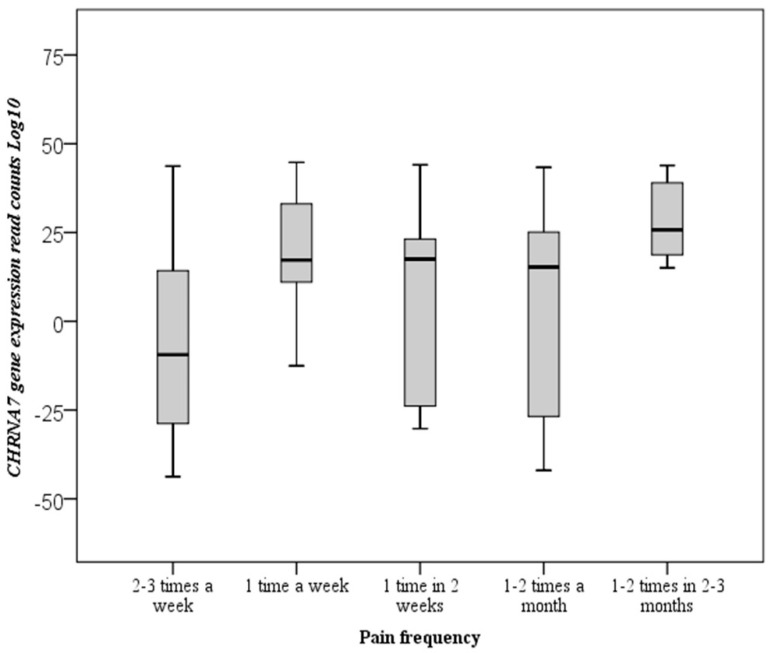
Boxplot of the *CHRNA7* gene expression for migraine frequency according to Dunn’s multiple comparison test results. The total numbers of mRNA reads are log10 transformed.

The *p*-value was calculated according to the categories by making multiple comparisons of pain frequency, by week and month, using Dunn’s test. The frequency of pain observed as 2–3 per week, which was previously affected by the decrease in gene expression level, also affected the association of pain frequency, which was 1 to 2 per week (*p* = 0.004), and 1–2 per 2–3 months (*p* = 0.003) in multiple comparisons. The frequency of pain was also found statistically significant in multiple analyses (Figure 2).

**Figure 3:**
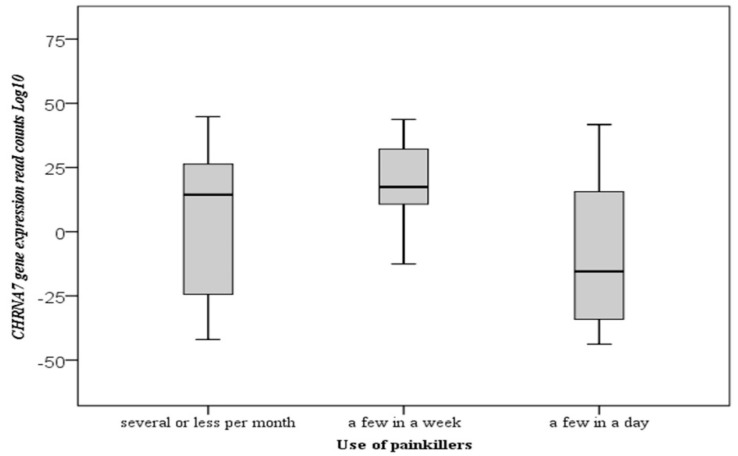
Boxplot of the *CHRNA7* gene expression for use of painkillers according to Dunn’s multiple comparison test results. The total numbers of mRNA reads are log10 transformed.

The *p*-value was calculated according to the categories by making multiple comparisons of the frequency of taking painkillers by the day, week, and month using Dunn’s test. It was found that the frequency of intake of painkillers was statistically significant in multiple analyzes regarding the frequency of taking a few or less per month (*p* = 0.035) and a few per week (*p* = 0.002) (Figure 3). As a result, downregulation of the gene affects the frequency of pain; individuals with the most severe pain (due to the most reduced expression of *CHRNA7*) are more prone to the frequent use of painkillers. The use and frequency of painkillers decrease gene expression, and other patient characteristics are not associated with gene expression.

### 3.6. The Relationship of CNV with Clinical Variables

The *p*-values were calculated by examining the relationship between clinical variables of migraine patients and CNV of the *CHRNA7* gene. The relationship among diabetes (*p* = 0.019), photophobia (*p* = 0.015), and CNV were found to be statistically significant (Table 7).

While there is no relationship between DM and *CHRNA7* gene expression levels, the significant relationship between DM and CNV of the gene suggests that it may be related due to the effect of not having diabetes. This result provides important evidence to exclude the effect of diabetes. Again, the relationship between copy number deletion and photophobia in patients with photophobia and a decreased CNV count suggests that photophobia may have arisen due to deletion. There was no relationship between other variables and CNV (*p* < 0.050). This result showed that clinical variables might affect expression, but do not have a direct effect on CNV.

### 3.7. The Receiver Operator Curve (ROC) Analyses

For the CHRNA7 gene expression, the area under the curve (AUC) was set to 0.731 (95% CI = 0.667–0.778) and the criterion was ≤19.79. Under these conditions, the sensitivity is 66.67% and the specificity was 80.83%. For the CNV, the AUC was set to 0.538 (95% CI = 0.470–0.605) and the criterion was >2. The sensitivity was 30.4% and the specificity was 79.0% When *CHRNA7* gene expression and CNV are analyzed together, the differences between areas are 0.194 and *p* < 0.001 (95% CI = 0.092–0.295) (Table 8).

## 4. Discussion

Family and twin studies, which are most commonly used to determine the basis of the disease and how close or far from social norms in genetics, have shown that there are genetic factors that cause migraine susceptibility. Variants with small effects, at more than one genetic locus, likely contribute to the clinical diversity of the disease; it is thought to be the effect of monogenic or polygenic inheritance. Genetic evidence based on migraine has been obtained from rare hereditary migraine disorders, such as hemiplegic and familial hemiplegic migraines, which can be caused by mutations in a single gene [28]. The genes responsible for the primary of these types include calcium voltage-gated channel subunit alpha 1 A (*CACNA1A*), ATPase Na^+^/K^+^ transporting subunit alpha 2 (*ATP1A2*), sodium voltage-gated channel alpha subunit 1 (*SCN1A*), proline-rich transmembrane protein 2 (*PRRT2*), casein kinase 1 delta (*CSNK1D*), three prime repair exonuclease 1 (*TREX1*), collagen type IV alpha 1 (*COL4A1*), and notch receptor 3 (*NOTCH3*) genes. In studies aiming to find the real relationships between migraines and genes, and comparing genome-wide association studies (GWAS) data and meta-analyses, various genes and pathways shown as therapeutic targets related to migraine susceptibility were identified [29]. Metabolic pathways, in which these genes are involved, and various polymorphisms and mutations of these genes, are also effective. In addition to the known genes, candidate gene studies have a large place in elucidating the complex nature of migraines.

Studies in patients with chronic migraines have shown that pro-inflammatory cytokine levels are increased in these patients. This is because the transcription of inflammatory genes is upregulated [30]. In vivo and in vitro experiments have shown that astrocyte and microglia activation accelerates the expression of pro-inflammatory cytokines, and related genes contribute to migraine, and microglial activation induces acute pain. In addition, both microglia and astrocytes were shown to participate in cortical spreading diseases associated with depression, such as migraines. Activation of the cholinergic anti-inflammatory pathway is a prophylactic approach to migraine. Studies show that α7nAChR contributes to neuronal protection. α7nAChR activation shows significant anti-inflammatory effects in neuroinflammation, and α7nAChR agonists have also been shown to attenuate hyperalgesic effects in chronic neuropathic pain [31].

Generally, SNP studies have been used to elucidate the genetic basis of migraine [32]. Undoubtedly, CNVs are increasingly the focus of important research in medical genetics, and their research has been particularly important in efforts to characterize the genetic basis of psychiatric and neurodevelopmental phenotypes. CNVs are generally defined as 1 kb or greater (smaller than 1 kb are considered indels) genomic segments that show copy number variation between individuals relative to the reference genome. Chromosome 15q13 is a hot spot for these CNVs due to the presence of low copy repeat (LCR) elements that facilitate non-allelic homologous recombination (NAHR). Many of these CNVs are overrepresented in individuals (with neuropsychiatric disorders. However, variable expression and incomplete penetration are common. It was suggested that the dosage sensitivity of the *CHRNA7* gene contributes greatly to the observed cognitive and behavioral phenotypes because it represents the smallest region of overlap for all 15q deletions and duplicates. Individuals with zero to four copies of CNVs in the *CHRNA7* gene have been reported in the literature, and deletions often represent a range of clinical severities leading to more severe and higher penetrating phenotypes. CNVs have been shown in many neuropsychiatric conditions, including autism spectrum disorder (ASD), schizophrenia, and mental disability (ID). CNVs in the 15q13.3 region showed incomplete penetrance, and deletions cause neuropsychiatric phenotypes in about 80% of cases, and most likely copies with lower penetration [16]. *CHRNA7* gene, which has one of these CNVs, plays a major role in the neuropsychiatric phenotypes observed in patients [33].

In this study, the status of CNVs in migraine disease and their effect on the expression of the gene containing CNVs were analyzed together with all clinical findings of the disease. Identifying the gene expression, which has an important place in the receptor mechanism mentioned before, and showing its relationship with the copy number, may reveal its effects on the genetics and pathology of migraine. Most importantly, it will be able to increase its potential as a therapeutic target. Among our reasons for choosing the gene is that it has therapeutic potential in the light of all this information, and it is predicted that the expression level, and CNV may be effective. In light of all this information, the reason why this gene is preferred is that it has therapeutic potential, and it is predicted that the expression level and CNV variation in the hotspot region may be effective in the pathology of the disease. In studies conducted on this subject, the detection of pain relief as a result of the effect of nicotine gum on the nAChRα7 receptor with applications showing the analgesic effect of nicotine gum strengthens our claim that the behavior of this receptor may be related to migraines. In applications showing the analgesic effect of nicotine gum, the detection of pain relief as a result of its effect on the nAChRa7 receptor strengthens our claim that the behavior of this receptor may be associated with migraine [23]. The expression data we obtained in our migraine patient group is important in that it is the first in the literature. Although its effect on many neurological and psychiatric diseases has been demonstrated, it has not been characterized in migraines. The regulation and function of gene expression are complex. There are different mechanisms involving partial duplication or deletion of the parent’s genes. α7nAchRs can be localized pre- and postsynaptically. It is also expressed in peripheral blood associated with various brain diseases and is usually downregulated [34]. It has been determined that patients with both deletion and duplication for *CHRNA7* gene CNVs exhibit a similar neuropsychiatric phenotype. This result indicates that most CNVs involve many genes and, thus, the neuropsychiatric phenotypes may be associated with other genes within the CNV.

In our migraine group, where peripheral blood samples were studied, the *CHRNA7* gene was downregulated as in other diseases in the studies mentioned. Its expression was significantly decreased in migraine patients, as it was found in whole blood. The loss of activity of the gene means that this effect of α7nAChR, which reduces the neuroinflammatory response in the normally activated state, is lost, leading to the formation of migraines. As a result, the decrease in the expression of the *CHRNA7* gene may contribute to the formation of migraines by inactivation of α7nAChR. Loss of the gene’s activity, which is activated in a normal process and has a reducing effect on the neuroinflammatory response, means that this effect of α7nAChR is lost and leads to migraine formation. In conclusion, decreased expression of *CHRNA7* gene may contribute to migraine by inactivation of α7nAChR.

When the clinical variables of migraine patients were examined, the number of individuals exposed to additional factors, such as smoking, diabetes, and hypertension, was very low in our group, and it was observed that they did not affect gene expression. It is known that attacks trigger headaches in smoking migraine patients. Based on this, it can be said that it does not affect gene expression, since the number of patients who smoked was also low, and it is an important point in patient selection to exclude this factor. In addition, migraines are associated with insulin resistance and type 2 diabetes. However, while investigating the cause of the disease in genetic studies, it was very important to exclude other underlying causes that would reduce the effect of the gene of interest. The number of patients with diabetes was six in our group, and it was not associated with gene expression. Migraines are a symptom of hypertension (hence, the association between migraines and hypertension). In addition, the inability to control uncontrolled blood pressure increases the frequency and severity of migraines [35]. Only seven of our patients had hypertension, which shows both the sterility of the group and the exclusion of this factor. Thus, it is not related to the downregulation of the gene. It has a strong genetic basis as well as the familial clustering of common migraine associated with (polygenic) polymorphisms in many genes, as well as rare migraine disorders caused by (monogenic) mutations. Where the genetic basis is precisely linked to a specific mutation or polymorphism, there is also the possibility of it being passed on to subsequent generations. In addition, when examined, in terms of migraine types, epidemiological studies showed that migraines with aura clustered more deeply in families than migraines without aura. In a study conducted to evaluate the heritability of common forms of migraines, the relationship between pediatric migraine patients with aura and their family histories was investigated. The family history of these patients was more likely than pediatric migraine patients without aura. In addition, it was shown that migraines occur at a younger age in children with parental history than children with a negative family history [36]. Although the genetic basis is still unknown, the findings of the above-mentioned studies highlight the heritability of migraines, which reveals the differences in inheritance patterns between migraines with aura and migraines without aura, and the age of onset between generations. It was also found that first-degree relatives had an increased risk of migraines with and without aura [37]. In the GWAS study, which included the determination of genes involved in the age of onset and pathology of migraines in Asians, one SNP was associated with 12 years of age and five SNPs were associated with migraines [38]. However, an important limitation of these studies is the patient grouping method, with grouping based on aura and family history. In our group, there were 60 individuals with family histories, and 42 individuals without. In addition, there was no relationship between *CHRNA7* gene expression and family history. This gene is not primarily responsible for migraines, but can have a multifactorial effect, and there are no definitive results, such as mutation and polymorphism showing its relationship with the disease, and it is an expected result that it is not associated with family history.

We know that the importance of gender in migraine patients is higher in females. As in all studies demonstrating the importance of gender in migraines, a significant relationship was found between migraines and gender in this thesis (*p* = 0.001). It is believed that female sex hormones play a major role in the development of migraines and possibly contribute to the sex differences observed in this disease. The prevalence rate of migraines can be seen, as it is associated with periods of major hormonal changes throughout life. Changes in the prevalence of migraines caused by fluctuations in hormones affect women disproportionately due to the nature of hormonal cycles. It is striking that gender is generally, in genetic studies, related to migraines, regardless of which gene is involved. Such a result shows that the frequency of migraines in women is due to sex hormone receptor binding rather than gene connection, differences in exposure to environmental stress, pain and stress response, and most importantly, differences in women’s life cycles (pre-menopause, post-menopause).

In the candidate gene and GWAS studies, conducted to reveal the migraine risk, quite a lot of gene variants were detected in migraines without aura, but definite results could not be obtained for migraines without aura. The limited SNP obtained in studies on migraines with aura only suggested that the two types of migraines may be similar in terms of genetic background [3]. To elucidate this point, it is important to understand the differences between the very common types of migraines. To detail migraines with aura, epigenetic markers with dynamic behavior could be used for dynamic mechanisms, such as attacks, and new candidate genes and protein-based targets can be searched with molecular modeling studies. In our study, there is no relationship between the type of migraine with and without aura, which consists of approximately equal numbers of individuals, and *CHRNA7* gene expression. The effect of genes is observed in a familial, monogenic, and polygenic way, rather than these two types of migraines. A hemiplegic migraine is a rare type of migraine with aura. Mutations in genes encoding ion channels and proteins cause the disease. Functional studies in cellular and animal models show that mutations that generally make the brain more susceptible to cortical spreading depression result in glutamatergic neurotransmission and cortical hyperexcitability, a phenomenon thought to coincide with aura symptoms. It is thought that there is no relationship between *CHRNA7* gene expression and types of migraines through these mechanisms and that there is no difference between the types of downregulation of the gene.

There is no relationship between gene expression and clinical findings, such as duration of pain, side, location, a form of onset, accompanying autonomy, phonophobia, photophobia, severity, MIDAS scale, presence (or absence) of provoked, mood, and equivalent in our migraine patients. These characteristics are not related to gene expression since the disease is not determinant, and they do not have a mechanism that works primarily on genes. The frequency of headache attacks ranges from approximately once a week or once a month, to once a year, for as little as an hour to three days in duration. The variability in the frequency of migraine attacks is related to both the genetic component carried by the individual and environmental trigger factors. In a study in which changes in RNA expression were measured during and outside migraine attacks, it was found that, due to changes in the gene, molecular mechanisms, such as transmembrane transport of ions, and response to stress are also changed. In another study, self-reported migraines and their complex causes were investigated; 18 potential factors, including environmental, occupational, and stress-related factors, were identified [39]. Gene expression fluctuates according to environmental and endogenous events and is, therefore, likely to elicit changes during a migraine attack. The fluctuation characteristic of gene expression over time enables a temporal design. That is, it follows changes in gene expression during the fluctuation of disease symptoms. Thus, the analysis of individual temporal changes makes it possible to examine the molecular mechanisms involved in the migraine attack. However, in multifactorial diseases, it is difficult to find the directly responsible gene and the changes during the attack. The findings here are that, in terms of the relationship between the frequency of the migraine and *CHRNA7* gene expression, it is observed that the gene expression is considerably reduced in individuals with a frequency of pain once or twice a week. Therefore, the link between the frequency of migraine and gene expression can be observed in this study. Significant reduction of gene expression may result from loss of function of mechanisms in the metabolic pathway. Gene duplication and deletion, which changes the dosage of the gene, is more prominent than mutations and polymorphisms on the *CHRNA7* gene. A rare deletion in the gene was associated with growth retardation and mental disability. Duplication of the gene was detected in two patients with autism and intellectual disability [40]. The 15q13.3 microdeletion, which is the site of the gene, is associated with neurodevelopmental phenotypes. The absence of a distinct mutation and polymorphism on the gene (which is often the cause of changes in the expression) suggests that the decrease in gene expression may be caused by other genetic and regulatory factors.

The effects of pain relievers on gene expression are more obvious. The *CHRNA7* gene expression level is very low in individuals using a few painkillers a day, and there is a strong relationship between these two parameters (*p* = 0.009). The type of pain reliever is as important as the frequency of use of painkillers. The combination of painkillers and anti-inflammatory drugs can alter gene expression at different levels.

Monogenic migraine disorders have a huge impact on individuals and families, but they are rare. The majority of migraines are polygenic, meaning it is a complex disease, in which multiple variables in genes contribute to the underlying risk, and each usually has a relatively small effect. The disease susceptibility is the result of these genetic variations interacting with each other, as well as with environmental and lifestyle factors. Discovering loci and genes that co-contribute to migraines requires different approaches to Mendelian disorders, mainly based on finding differences in allele frequencies of gene-linked genetic variants, which are comprised of unrelated individuals, between cohorts of migraine cases and non-migraine controls. Common genetic variations consist largely of SNPs, small insertion (insertion) or deletion (deletion), short tandem repeats, and copy number variants. Most efforts to identify variants that affect characteristics and disorders, including migraine, have focused on SNPs that provide increased or decreased risks of migraines. Effect sizes are usually small for most loci. It requires the genotyping of large numbers of individuals to robustly obtain results that exceed significance thresholds [41]. A filtering process is then required to narrow down the number of variants. This can be done in several ways. They all depend on several existing databases containing genetic information. These databases consist of collections of structural variants, such as SNPs and CNV variants detected by different studies. Hot spots for variants are locations that are closely related to the disease. CNVs are a common source of structural genomic variation that include gains (copies or insertions), losses (deletions), or complex rearrangements of the genomic sequence that cause deviations from the diploid state. On average, each individual is estimated to have >1000 CNV and contain ~10 megabases (Mb) genomic sequence, with similar gain and loss rates [42]. When these data are obtained from family-based studies, the behavior of the disease can be revealed by following the risky variants, their transmission to generations, and their transmission characteristics. In addition, CNVs have been shown to contribute to migraines in twin studies [5].

In our migraine patients, a relationship was found between the decreased copy number and increasing copy number compared to healthy control individuals and the risk of migraines. CNVs can affect the phenotype and directly cause disease by disrupting the function of genes and/or changing the gene dosage [19]. In addition, CNVs can indirectly affect gene expression through position effects by changing the communication between alleles, by unmasking the recessive mutation or deleting regulatory elements. For clinical use, each CNV detected must be interpreted and distinguished from pathogenic or high-risk benign variants. However, it should be emphasized that the interpretation of CNVs is highly dependent on clinical indications. Clinicians must provide sufficiently detailed clinical phenotypes to ensure correct interpretation of the results. When genes are reported as pathogenic in the literature, it is important to investigate the nature of variants. Copy number gain of the gene associated with the clinical phenotype due to the mutation may be due to a different clinical picture. Disorders are usually caused by the acquisition of function mutations rather than dosage imbalance. Thus, CNVs containing such genes may not have clinical significance or may result in a completely different phenotype (e.g., mutations in *FGFR1* lead to skeletal dysplasia while deletions are associated with Kallmann syndrome) [43].

Copy number gains involving only part of a gene can result in disruption of the gene or alteration of the coding sequence [44]. Deletion of genes associated with recessive diseases may indicate a mutation on the second copy of the gene. Small variants containing only intronic sequences may still affect gene function. However, when the genes within the detected CNV are not reported as pathogenic in the literature, and the role of the gene relies solely on the predicted gene function or function characterized in model organisms, conclusions regarding pathogenicity cannot be drawn until the variant is well characterized in the human population. In addition, CNV frequencies also differ between populations. It is considered rare when its frequency is lower than 1%, and commonly when it is higher than 1%. Both types of CNVs can occur in the normal population as well as in patients with the abnormal phenotype [45]. CNV in our migraine patients displays a gain + loss subtype located on chromosome 15q13.3. Studies on gains point to micro-duplications. These gains were found in diseases such as autism spectrum disorder, bipolar, anxiety, as well as various developmental retardation or mental retardation cases [16].

The direct relationship of this CNV in the *CHRNA7* gene with diseases is not clear. However, clinical phenotypes can customize this relationship. According to the results of this study, we can say that increased and decreased copy numbers may be associated with migraine. Either way, due to the change in the dosage of the gene, its function will also change. Since the product of the gene is a receptor function, the decrease in the expression of the receptor causes deregulation for ion passage. When evaluated in terms of clinical phenotypes, smoking is not associated with the number of CNVs. Smoking is known to affect gene expression. Studies are showing that smoking changes CNV when smoking is evaluated together with various diseases. The relationship is linked to a large number of copies. In our migraine group, CNV is not associated with smoking, since the number of individuals with reduced copy numbers is high, and the number of individuals with increased copy numbers is low. Although diabetes is associated with CNV, its type is also important. It is also thought to be a predictive sign. The copy numbers of studies for risky genes in type-2 diabetes patients have shown that epidermal growth factor receptor (*EGFR*), E2F transcription factor 1 (*E2F1*), protein phosphatase 1 regulatory subunit 3A (*PPP1R3A*), human leukocyte antigen (*HLA*), and tetraspanin 8 (*TSPAN8*) are important. Some of the genes are responsible for the control of the cycle of pancreatic exocrine cells, some for the regulation of the sustainability of pancreatic beta cells, and type 2 diabetes mellitus **(***T2DM*) is responsible for corneal pathology in diabetic patients [46]. CNVs may be associated with different clinical findings in multifactorial disease. The screening was performed for microdeletion and micro-duplication in the 15q13.3 region of the *CHRNA7* gene CNVs in induced pluripotent stem cells and neural progenitor stem cells. Many diseases, including diabetes, have been associated with receptor-dependent errors in the calcium intake mechanism [47]. The fact that patients in our migraine group did not have a history of diabetes, other than six individuals, is important, in terms of clearly demonstrating the effect of CNV count on the disease and excluding a factor that may be a factor. In our results, there was no effect that diabetes may have brought, and its significance in migraine patients with decreasing and increasing numbers suggests that it may be protective of diabetes (*p* = 0.019). For hypertension and CNV linkage, CNV belonging to this gene has not been studied in direct hypertension patients, but some results include cases of association with other diseases or losses of CNVs associated with other genes. In our migraine group, the absence of a history of hypertension where CNVs are affected is important in terms of excluding its effect, and it is an expected result that there is no relationship between CNV and the risk of migraine. CNVs are defined as changes in the copy numbers of large genomic segments that can be inherited from parents or that occur de novo [48]. Copy number variation is caused by the copying or deletion of a gene segment within a particular chromosome. However, in many cases, deletion and duplication can be found in the same location. When the CNVs are associated with the disease, if there is a germ-line change, it is possible to be transferred to the next generations. CNVs differ between individuals. This situation is called CNV polymorphism. A CNV can occur (de novo or inherited). Familial CNVs provide information about transmission and the nature of the disease. In our migraine group, there were 40 individuals with a family history and 32 individuals without a family history. The absence of a relationship suggests that there is no familial transmission for this CNV studied. When the relationship between migraine types and CNV is examined, individuals with aura constitute about half of those without aura. The fact that CNV is not related to migraine types may be due to the numerical differences of patient types. There are no studies in the literature regarding CNV and migraine forms. Considering that the number of copy deletions or duplications is multifactorial of migraine, and is not related to parameters such as duration, frequency, the severity of pain, MIDAS scale, mood, equivalent, use of painkillers, and location, it can be said that it is not associated with migraine forms, as it does not exhibit a clinical independent genetic link. Photophobia, which is among the clinical findings of migraines, is not only a sensitivity alone, but is also associated with diseases, such as associated macular degeneration [49].

The presence of eye defects was not questioned in our migraine group. However, it is thought that the number of severe visual disorders accompanying migraines in the group of 102 people is not likely high enough to affect the significance. Therefore, when the number of individuals with photophobia complaints in migraine patients is examined, the correlation between the decrease and increase in the number of CNV and photophobia must be the first finding in the literature. Photophobia is one of the symptoms of migraines. It is one of the most important criteria indicating migraines in headache classification. There is no study in the literature investigating the connection between photophobia alone and CNVs. Light is carried by visual pathways to the brain through the retina. The retina has cones, the cells responsible for vision, and a melanopsin system that does not participate in vision, but perceives light. These cells connect to the trigeminal system in the brain centers and, thus, can cause pain. The activation of neurons that carry the signal from the spinal cord to the thalamus, called secondary neurons, can be modulated by α7nACh receptors, which act presynaptically in the transmission of nociceptive information to the central nervous system. Besides peripheral sensitization, the continuous activation of second-order trigeminal neurons translates to central sensitization in migraineurs when non-nociceptive stimuli produce pain. Glutamate receptor activation is added to this sensitization. Further activation and sensitization of the trigeminal system may result in the sensitization of third-order neurons that carry the signal from the thalamus to the primary sensory cortex. This condition leads to other migraine symptoms, including photophobia, phonophobia, and osmophobia. Based on the ROC analyzes reported, *CHRNA7* gene expression is thought to have potential as a putative regulator or a biomarker of migraine sensitivity. The loss and gain of CNV count for the *CHRNA7* gene, which is observed to have an important place in this pathway, is thought to contribute to the symptoms of photophobia in patients related to the loss of function of these receptors.

When the effect of CNV on the gene on the expression level of the *CHRNA7* gene was examined, a statistically significant relationship was not observed. The gene expression level in individuals with the decreased copy number in migraine patients was significantly lower than in the control. Again, the expression level in migraine patients with the increased copy number was much lower than in the control. The results indicate that there may be a link between the gain and loss of the *CHRNA7* gene in the CNV region and the expression of the gene in migraine patients. Increasing the number of groups can help reveal the link. Differences in the DNA sequence of our genomes increase our uniqueness. These changes affect many traits, including disease susceptibility. Since CNVs generally involve genes, they can have important roles in both human diseases and drug response, and revealing their link with diseases is important for both treatment processes and correction of mechanisms of occurrence.

## Figures and Tables

**Table 1:** Patient and control characteristics of the different cohorts.

Characteristics	Control (%) (*n* = 120)	Migraine (%) (*n* = 102)
**Age (years)**		
15–20	5 (4.2)	6 (5.8)
21–25	16 (13.3)	11 (10.8)
26–30	13 (10.9)	20 (19.6)
31–35	24 (20)	22 (21.6)
36–40	31 (25.8)	13 (12.7)
41–45	22 (18.3)	14 (13.7)
46–50	9 (7.5)	12 (11.8)
51–55	0	2 (2)
56–60	0	2 (2)
**Gender (*n*, %)**		
Female	73 (60.8)	90 (88.2)
Male	47 (39.2)	12 (11.8)
**Migraine type**		
with aura	NA	43 (42.2)
without aura		59 (57.8)
**Headache frequency (hour)**		
less than 12	NA	26 (25.5)
12–24		35 (34.3)
24–48		29 (28.4)
48–72		12 (11.8)
**Site of pain**		
unilateral	NA	52 (51)
bilateral		32 (31.4)
unclear		18 (17.6)
**Location of pain**		
from the neck	NA	42 (41.2)
from the temples		43 (42.2)
no localized		17 (16.7)
**Starting of pain**		
generally sudden and severe	NA	32 (31.4)
usually starts mild and becomes severe		62 (60.8)
not clear		8 (7.8)
**Autonomic symptoms**		
**(hot flashes, sweating, chills, diarrhea, abdominal pain, nausea, high blood pressure)**		
accompanies	NA	73 (71.6)
not accompanies		29 (28.4)
**Phonophobia**		
always	NA	76 (74.5)
sometimes		19 (18.6)
absent		7 (6.9)
**Photophobia**		
always	NA	70 (68.6)
sometimes		26 (25.5)
absent		6 (5.9)
**Severity**		
very severe	NA	45 (44.1)
severe		43 (42.2)
mild		10 (9.8)
moderate		4 (3.9)
**Frequency of headache**		
2–3 times a week	NA	32 (31.4)
1 time a week		23 (22.5)
1 in 2 weeks		9 (8.8)
1–2 times a month		30 (29.4)
1–2 times in 2–3 months		8 (7.8)
**MIDAS**		
0–5, little disability	NA	40 (39.2)
6–10, mild disability		21 (20.6)
11–20, moderate disability		35 (34.3)
20+, severe disability		6 (5.9)
**Trigger factors**		
**(irritability, fatigue, insomnia, menstruation, nutritional status, fasting period)**		
available	NA	85 (83.3)
not available		17 (16.7)
**Mood**		
available	NA	82 (80.4)
not available		20 (19.6)
**Equivalent**		
available	NA	35 (34.3)
not available		67 (65.7)
**Alcohol**		6 (5.9)
**Cigarette smoke**		24 (23.5)
**Use of painkillers**		
several or less per month	NA	40 (39.2)
a few a week		37 (36.3)
a few a day		23 (22.5)
4 per day		2 (2)
**Disease history**		
no history	97 (80.8)	72 (70.6)
Hypertension	7 (5.8)	5 (4.9)
chronic lung disease	1 (0.8)	2 (2)
Kidney disease	1 (0.8)	2 (2)
Diabetes	5 (4.2)	5 (5)
Infections	0	0
Stroke	0	0
Endocrine disease	3 (2.6)	6 (5.9)
Asthma	2 (1.7)	2 (2)
Hypertension + chronic lung disease	2 (1.7)	0
Hypertension + diabetes	1 (0.8)	1 (0.8)
Hypertension+ infection	1 (0.8)	1 (0.6)
Hypertension + endocrine	0	1 (0.8)
Unclassified	0	6 (6)
Family History	0	9 (8.8)

MIDAS: migraine disability assessment.

**Table 2:** Blood parameters between the groups.

Parameters	Control (%) (*n* = 120)	Migraine (%) (*n* = 102)	*p*-Value
Age	34.52 ± 7.79	34.39 ± 10.03	0.703
BMI	25.11 ± 4.24	25.46 ± 4.45	0.575
WBC	8363.84 ± 3008.62	7446.13 ± 2730.98	0.025 *
Urea	30.35 ± 1.55	29.03 ± 12.14	0.275
GFR	103.13 ± 17.93	105.58 ± 10.58	0.703
ESR	14.97 ± 8.72	19.52 ± 11.73	0.007 *
CRP	4.06 ± 2.63	3.79 ± 2.62	0.466
HBG	12.86 ± 2.21	12.14 ± 2.29	0.027 *

*p*-values < 0.05 were considered significant for variables between migraine patients and control samples. * Boldface indicates statistically significant according to the Mann–Whitney *u*-test. BMI: body mass index, WBC: white blood cell, GFR: glomerular filtration rate, ESR: erythrocyte sedimentation rate, CRP: C-reactive protein, HBG: hemoglobin.

**Table 3:** *CHRNA7* gene expression, minimum, maximum, and *p* values in migraine patient and control group.

Gene	Group	*n*	Mean ± SD	Median	25th	75th	OR (%95 CI)	*p*-Value
***CHRNA7***	Control	120	27.45 ± 8.78	28.09	10.19	−44.39	1 (reference)	0.001 *
	Migraine	102	6.44 ± 27.29	12.85	−43.78	−44.78	0.94 (0.92–0.96)	

* *p*-values < 0.05 were considered significant, *n*: number of individuals, OR: odds ratio, CI: confidence interval, SD: standard deviation, reference: *ACTB* gene; boldface indicates statistically significant according to Mann–Whitney *u*-test. Values are presented as median (25th and 75th percentile).

**Table 4:** *CHRNA7* gene CNV and *p*-values in migraine patients and control group.

CNV	Groups		OR (95% CI)	*p*-Value
	Control *n* (%)	Migraine *n* (%)		
Loss (<2)	30 (25.0)	72 (70.6)	23.66 (9.75–57.41)	0.001 *
Normal (=2)	69 (57.5)	7 (6.9)	1 (Reference)	
Gain (>2)	21 (17.5)	23 (22.5)	10.79 (4.06–28.68)	0.001 *

* *p*-values < 0.05 were considered significant, *n*: number of individuals, OR: odds ratio, CI: confidence interval, reference: *TERT*. Boldface indicates statistically significant according to Chi-square test.

**Table 5:** Mean, median, and *p*-values of CHRNA7 gene expression and CNV in migraine patients and control group.

Group	CNV	*n*	Gene Expression				*p*-Value
			Mean ± SD	Median	25th	75th	
**Control**	Loss (<2)	30	27.73 ± 9.96	27.99	10.43	44.39	0.981
	Normal (=2)	69	27.22 ± 7.67	28.2	10.33	43.97	
	Gain (>2)	21	27.83 ± 10.71	27.71	10.19	43.55	
**Migraine**	Loss (<2)	72	6.49 ± 27.94	12.82	−43.78	44.78	0.940
	Normal (=2)	7	5.98 ± 28.2	19.05	−30.08	36.99	
	Gain (>2)	23	6.42 ± 26.14	12.77	−43.21	43.38	

*p*-values < 0.05 were considered significant, *n*: number of individuals, SD: standard deviation. Boldface indicates statistically significant according to Chi-square test. Values are presented as median (25th and 75th percentile).

**Table 6:** Association of clinical characteristics and *CHRNA7* gene expression.

Cohorts	*n*	Mean ± SD	Median	25th	75th	*p*-Value
**Cigarette smoke**						0.776
Yes	24	6.36 ± 27.59	13.58	−35.77	44.53	
No	78	6.46 ± 27.38	12.82	−43.78	44.78	
**Diabetes**						0.926
Yes	6	4.78 ± 24.8	14.76	−28.04	32.64	
No	96	6.54 ± 27.55	12.85	−43.78	44.78	
**Hypertension**						0.385
Yes	7	13.84 ± 26.63	16.50	−24.40	44.78	
No	95	5.89 ± 27.4	12.77	−43.78	44.53	
**Migraine attack**						0.336
Yes	60	4.47 ± 27.21	11.94	−43.21	44.53	
No	42	9.26 ± 27.48	14.83	−43.78	44.78	
**Migraine type**						0.085
with aura	43	2.08 ± 25.42	12.12	−43.78	41.72	
without aura	59	9.61 ± 28.37	15.03	−43.21	44.78	
**Headache frequency**						0.682
**less than 12 h**	26	6.29 ± 24.28	13.25	−30.08	41.70	
12–24 h	35	10.13 ± 28.68	17.91	−43.78	44.78	
24–48 h	29	4.57 ± 30.03	12.01	−43.21	44.53	
48–72 h	12	0.48 ± 23.84	11.64	−30.23	41.19	
**Site of pain**						0.892
unilateral	52	5.61 ± 27.58	12.08	−43.21	44.53	
bilateral	32	6.91 ± 27.89	15.57	−43.78	44.78	
unclear	18	8.01 ± 26.81	13.58	−37.80	43.71	
**Location of pain**						0.204
from the neck	42	10.34 ± 24.49	17.57	−36.3	44.53	
from the temples	43	5.76 ± 29.61	12.12	−43.78	44.78	
no localized	17	−1.49 ± 27.47	11.31	−43.21	43.71	
**Starting of pain**						0.861
generally sudden and severe	32	5.34 ± 26.33	12.57	−43.17	43.71	
usually starts mild and becomes severe	62	6.88 ± 27.52	13.66	−43.78	44.78	
Not clear	8	7.44 ± 32.67	18.81	−35.76	41.72	
**Autonomic symptoms**						0.124
accompanies	29	13.17 ± 24.79	17.49	−36.3	44.08	
not accompanies	73	3.76 ± 27.93	12.03	−43.78	44.78	
**Phonophobia**						0.379
always	76	8.38 ± 25.94	13.35	−43.78	44.78	
sometimes	19	3.88 ± 32.04	17.49	−43.21	43.71	
absent	7	−7.72 ± 27.28	−23.88	−32.46	32.64	
**Photophobia**						0.530
always	70	4.72 ± 27.22	12.39	−43.78	44.53	
sometimes	26	10.81 ± 27.66	18.03	−33.48	44.78	
absent	6	7.53 ± 29.14	17.08	−32.44	38.66	
**Severity**						0.752
very severe	45	4.46 ± 29.01	12.65	−43.21	44.78	
severe	43	6.26 ± 25.34	12.03	−43.17	44.53	
mild	10	11.17 ± 32.80	25.35	−43.78	43.88	
moderate	4	18.87 ± 13.28	13.25	10.31	38.66	
**Frequency of headache**						0.008 *
2–3 times a week	32	−5.93 ± 27.12	−9.43	−43.78	43.71	
1 time a week	23	16.42 ± 24.11	17.21	−35.76	44.78	
1 time in 2 weeks	9	8.13 ± 27.62	17.49	−30.23	44.08	
1–2 times a month	30	6.95 ± 26.78	15.29	−41.99	43.38	
1–2 times in 2–3 months	8	23.41 ± 21.4	25.77	−23.49	43.88	
**MIDAS**						0.260
0–5, little disability	40	10.65 ± 26.8	17.78	−41.99	44.08	
6–10, mild disability	21	8.55 ± 27.62	14.63	−35.76	44.78	
11–20, moderate disability	35	2.82 ± 26.39	11.50	−43.21	43.72	
20+, severe disability	6	−7.94 ± 33.95	−14.88	−43.78	44.53	
**Trigger factors**						0.625
Yes	85	7.23 ± 26.19	12.92	−43.78	44.53	
No	17	2.51 ± 32.85	11.85	−43.17	44.78	
**Mood**						0.153
Yes	82	4.70 ± 27.70	12.08	−43.78	44.53	
No	20	13.57 ± 24.91	17.57	−41.99	44.78	
**Equivalent**						0.891
Yes	35	5.68 ± 28.51	14.63	−43.17	44.53	
No	67	6.83 ± 26.84	12.71	−43.78	44.78	
**Use of painkillers**						0.009 *
several or less per month	41	6.07 ± 28.07	14.39	−41.99	44.78	
a few a week	38	15.72 ± 21.47	17.43	−28.92	43.72	
a few a day	23	−8.23 ± 28.93	−15.48	−43.78	41.72	

*p*-values < 0.05 were considered significant for variables between migraine patient clinical characteristics and *CHRNA7* gene expressions. * Boldface indicates statistically significant according to Mann–Whitney U and Kruskal–Wallis test. NA: not applicable, MIDAS: migraine disability assessment. Values are presented as median (25th and 75th percentile).

**Table 7:** Evaluation of clinical variables and measurements of *CHRNA7* gene CNV.

Cohorts	CNV			*p*-Value
	Loss (<2) *n* (%)	Normal (= 2) *n* (%)	Gain (>2) *n* (%)	
**Cigarette smoke**				
Yes	20 (27.8)	1 (14.3)	3 (13.0)	0.292
No	52 (72.2)	6 (85.7)	20 (87.0)	
**Diabetes**				
Yes	4 (5.6)	2 (28.6)	0 (0.0)	0.019 *
No	68 (94.4)	5 (71.4)	23 (100.0)	
**Hypertension**				
Yes	4 (5.6)	1 (14.3)	2 (8.7)	0.632
No	68 (94.4)	6 (85.7)	21 (91.3)	
**Migraine attack**				0.491
Yes	40 (55.6)	4 (57.1)	16 (69.6)	
No	32 (44.4)	3 (42.9)	7 (30.4)	
**Migraine type**				
with aura	29 (40.3)	4 (57.1)	10 (43.5)	0.682
without aura	43 (59.7)	3 (42.9)	13 (56.5)	
**Headache frequency**				
less than 12 h	20 (27.8)	2 (28.6)	4 (17.4)	
12–24 h	24 (33.3)	1 (14.3)	10 (43.5)	0.681
24–48 h	21 (29.2)	3 (42.9)	5 (21.7)	
48–72 h	7 (9.7)	1 (14.3)	4 (17.4)	
**Site of pain**				0.436
unilateral	39 (54.2)	2 (28.6)	11 (47.8)	
bilateral	22 (30.6)	4 (57.1)	6 (26.1)	
unclear	11 (15.3)	1 (14.3)	6 (26.1)	
**Location of pain**				0.135
from the neck	26 (36.1)	4 (57.1)	12 (52.2)	
from the temples	35 (48.6)	3 (42.9)	5 (21.7)	
no localized	11 (15.3)	0 (0.0)	6 (26.1)	
**Starting of pain**				
generally sudden and severe	22 (30.6)	2 (28.6)	8 (34.8)	
usually starts mild and becomes severe	44 (61.1)	5 (71.4)	13 (56.5)	0.923
Not clear	6 (8.3)	0 (0.0)	2 (8.7)	
**Autonomic symptoms**				
accompanies	18 (25.0)	2 (28.6)	9 (39.1)	0.425
does not accompany	54 (75.0)	5 (71.4)	14 (60.9)	
**Phonophobia**				
always	53 (73.6)	5 (71.4)	18 (78.3)	
sometimes	15 (20.8)	0 (0.0)	4 (17.4)	0.149
absent	4 (5.6)	2 (28.6)	1 (4.3)	
**Photophobia**				
always	48 (66.7)	3 (42.9)	19 (82.6)	
sometimes	22 (30.6)	2 (28.6)	2 (8.7)	0.015 *
absent	2 (2.8)	2 (28.6)	2 (8.7)	
**Severity**				
very severe	31 (43.1)	4 (57.1)	10 (43.5)	
severe	30 (41.7)	3 (42.9)	10 (43.5)	0.667
mild	9 (12.5)	0 (0.0)	1 (4.3)	
moderate	2 (2.8)	0 (0.0)	2 (8.7)	
**Frequency of headache**				
2–3 times a week	21 (29.2)	2 (28.6)	9 (39.1)	
1 time a week	20 (27.8)	0 (0.0)	3 (13.0)	0.243
1 time in 2 weeks	4 (5.6)	1 (14.3)	4 (17.4)	
1–2 times a month	23 (31.9)	3 (42.9)	4 (17.4)	
1–2 times in 2–3 months	4 (5.6)	1 (14.3)	3 (13.0)	
**MIDAS**				
0–5, little disability	27 (37.5)	5 (71.4)	8 (34.8)	0.277
6–10, mild disability	14 (19.4)	1 (14.3)	6 (26.1)	
11–20, moderate disability	26 (36.1)	0 (0.0)	9 (39.1)	
20+, severe disability	5 (6.9)	1 (14.3)	0 (0.0)	
**Trigger factors**				
Yes	61 (84.7)	6 (85.7)	18 (78.3)	0.758
No	11 (15.3)	1 (14.3)	5 (21.7)	
**Mood**				
Yes	55 (76.4)	7 (100.0)	20 (87.0)	0.216
No	17 (23.6)	0 (0.0)	3 (13.0)	
**Equivalent**				
Yes	26 (36.1)	4 (57.1)	5 (21.7)	0.189
No	46 (63.9)	3 (42.9)	18 (78.3)	
**Use of painkillers**				
several or less per month	27 (37.5)	4 (57.1)	10 (43.5)	0.821
a few a week	27 (37.5)	2 (28.6)	9 (39.1)	
a few a day	18 (25.0)	1 (14.3)	4 (17.4)	

*p*-values < 0.05 were considered significant for variables between migraine patient clinical characteristics and CNV. * Boldface indicates statistically significant according to the Chi-Square test. MIDAS: migraine disability assessment.

**Table 8:** The receiver operating characteristics curve analyses of the expression of *CHRNA7* and CNV.

Compared Groups	Variation	AUC	95% CI	*p*-Value	Specificity (%)	Sensitivity (%)	Criterion
Migraine patients vs. Control	*CHRNA7* expression	0.731	(0.667–0.788)	<0.001	80.8	66.7	≤19.79
	CNV	0.538	(0.470–0.605)	0.2563	79.0	30.4	>2
AUC, area under the curve; CI, confidence interval.							

## Data Availability

Data are available upon request from the authors.

## Abbreviations

α7nAchR	alpha-7 nicotinic receptor
ACTB	Beta-actin
ADHD	Attention Deficit Hyperactivity Disorder
ASDs	Autism spectrum disorder
ATP1A2	ATPase Na+/K+ Transporting Subunit Alpha 2
AUC	Area under the curve
BMI	Body Mass Index
CACNA1	Calcium Voltage-Gated Channel Subunit Alpha1 A
*CHRNA7*	Cholinergic Receptor Nicotinic Alpha 7 Subunit
CNV	Copy Number Variation
COL4A1	Collagen type IV alpha 1
CRP	C-Reactive Protein
CSNK1D	Casein Kinase 1 Delta
E2F1	E2F Transcription Factor 1
EGFR	Epidermal Growth Factor Receptor
ESR	Erythrocyte Sedimentation Rate
GWAS	Genome-Wide Association Studies
HBG	Hemoglobin
HLA	Human Leukocyte Antigen
ICHD-3	The International Classification of Headache Disorders 3rd Edition
ID	Mental disability
LCR	Low Copy Repeat
NAHR	Non-allelic homologous recombination
NOTCH3	Notch Receptor 3
PPP1R3A	Protein Phosphatase 1 Regulatory Subunit 3A
PRRT2	Proline-rich transmembrane protein 2
SCN1A	Sodium Voltage-Gated Channel Alpha Subunit 1
SNP	Single Nucleotide Polymorphism
T2DM	Type 2 diabetes mellitus
TERT	Telomerase Reverse Transcriptase
TREX1	Three Prime Repair Exonuclease 1
TSPAN8	Tetraspanin 8
WBC	White Blood Cell
